# Global trends and collaborative networks in gut microbiota-insulin resistance research: a comprehensive bibliometric analysis (2000–2024)

**DOI:** 10.3389/fmed.2024.1452227

**Published:** 2024-08-15

**Authors:** Gulshara Zh Abildinova, Valeriy V. Benberin, Tamara A. Vochshenkova, Alireza Afshar, Nadiar M. Mussin, Asset A. Kaliyev, Zhanna Zhussupova, Amin Tamadon

**Affiliations:** ^1^Gerontology Center, Medical Center Hospital of the President’s Affairs Administration of the Republic of Kazakhstan, Astana, Kazakhstan; ^2^Corporate Foundation, Institute of Innovative and Preventive Medicine, Astana, Kazakhstan; ^3^Student Research Committee, Bushehr University of Medical Sciences, Bushehr, Iran; ^4^PerciaVista R&D Co., Shiraz, Iran; ^5^Department of Surgery No. 2, West Kazakhstan Medical University, Aktobe, Kazakhstan; ^6^Department of Neurology, Psychiatry and Narcology, West Kazakhstan Marat Ospanov Medical University, Aktobe, Kazakhstan; ^7^Department of Natural Sciences, West Kazakhstan Marat Ospanov Medical University, Aktobe, Kazakhstan; ^8^Stem Cells Technology Research Center, Shiraz University of Medical Sciences, Shiraz, Iran

**Keywords:** gastrointestinal microbiome, insulin resistance, insulin sensitivity, gut microbiota, short-chain fatly acids

## Abstract

**Background:**

The human gut microbiota plays a crucial role in maintaining metabolic health, with substantial evidence linking its composition to insulin resistance. This study aims to analyze the global scholarly contributions on the relationship between intestinal microbiota and insulin resistance from 2000 to 2024.

**Methods:**

A bibliometric analysis was conducted using data from Scopus and Web of Science Core Collection. The search strategy included terms related to “Gastrointestinal Microbiome” and “Insulin Resistance” in the title or abstract.

**Results:**

The analysis of 1,884 relevant studies from 510 sources was conducted, revealing a mean citation of 51.36 per manuscript and a remarkable annual growth rate of 22.08%. The findings highlight the significant role of gut microbiota in insulin resistance, corroborating prior studies that emphasize its influence on metabolic disorders. The literature review of the current study showed key mechanisms include the regulation of short-chain fatty acids (SCFAs) and gut hormones, which are critical for glucose metabolism and inflammation regulation. The analysis also identifies “Food and Function” as the most productive journal and Nieuwdorp M. as a leading author, underscoring the collaborative nature of this research area.

**Conclusion:**

The consistent increase in publications in the field of gut microbiota and insulin resistance indicates growing recognition of the gut microbiota’s therapeutic potential in treating insulin resistance and related metabolic disorders. Future research should focus on standardizing methodologies and conducting large-scale clinical trials to fully realize these therapeutic possibilities.

## 1 Introduction

The intestinal microbiota, a complex community of microorganisms residing in the human gut, plays a pivotal role in maintaining health and homeostasis (1, 2). It is estimated that the human gut hosts approximately 100 trillion microorganisms, most of them bacteria, but also viruses, fungi, and protozoa (1). The microbiome, the collective genomes of these microorganisms, encodes over 3 million genes producing thousands of metabolites, which replace many of the functions of the host, consequently influencing the host’s fitness, phenotype, and health (1, 3). The gut microbiota assists in a range of bodily functions, including harvesting energy from digested food, protecting against pathogens, regulating immune function, and strengthening biochemical barriers of the gut and intestine (4, 5). It is estimated that the gut microbiota produces thousands of metabolites that interact with the host and influence various physiological processes, including energy metabolism, immune response, and hormone regulation (3, 6). The gut microbiota is also implicated in the development of metabolic diseases, including insulin resistance, which is a key factor in the pathogenesis of type 2 diabetes and metabolic syndrome (7).

Insulin resistance is a condition in which the body’s cells become less responsive to insulin, leading to impaired glucose uptake and increased glucose levels in the blood (8). It is a major risk factor for the development of type 2 diabetes and metabolic syndrome, and is often associated with obesity, physical inactivity, and a sedentary lifestyle (8, 9). The gut microbiota has been identified as a key factor in the development of insulin resistance, as it regulates the production of metabolites that influence glucose and lipid metabolism (9). The gut microbiota also plays a role in the regulation of inflammation, which is a key mechanism underlying insulin resistance in adolescence and even children (10, 11).

The gut microbiota influences insulin resistance through several mechanisms (9). Firstly, it regulates the production of short-chain fatty acids (SCFAs), which are produced by the fermentation of dietary fiber and play a key role in regulating glucose and lipid metabolism (12, 13). Secondly, the gut microbiota influences the production of gut hormones, such as glucagon-like peptide-1 (GLP-1), which plays a key role in regulating glucose homeostasis and insulin secretion (14). Finally, the gut microbiota regulates the production of cytokines, such as tumor necrosis factor-alpha (TNF-alpha), which plays a key role in regulating inflammation and insulin resistance (15, 16). All of these studies suggested the important role of gut microbiota an its relationship with insulin resistance.

Hence, the scrutiny of the published literature on the relationship between gut microbiota and insulin resistance is vital for the formulation of novel empirical and therapeutic guidelines for diabetes treatment (17, 18). Moreover, the examination of the published literature is indispensable for comprehending the worldwide and regional shifts in diabetes. Bibliometric analysis emerges as a potent methodology in the quantitative assessment of academic papers, facilitating researchers to trace the evolution of specific fields; it is a beneficial instrument that utilizes mathematical and statistical techniques to evaluate the expansion, productivity, and overall patterns of publications pertinent to a specific topic (19–21). In the realm of medicine, such analyses assume a critical role in depicting research trends and advancements, inspiring researchers to pinpoint leading countries and institutions, as well as areas that necessitate enhancement. This study will act as a fundamental information source for future comparisons and evaluations. It aspires to identify trends and progress in this research area and illuminate potential areas for further concentration and improvement. In the present study, the significance of the relationship between gut microbiota and insulin resistance will be assessed through a bibliometric analysis of existing literature.

## 2 Methods and materials

### 2.1 Research strategy

**Figure 1:**
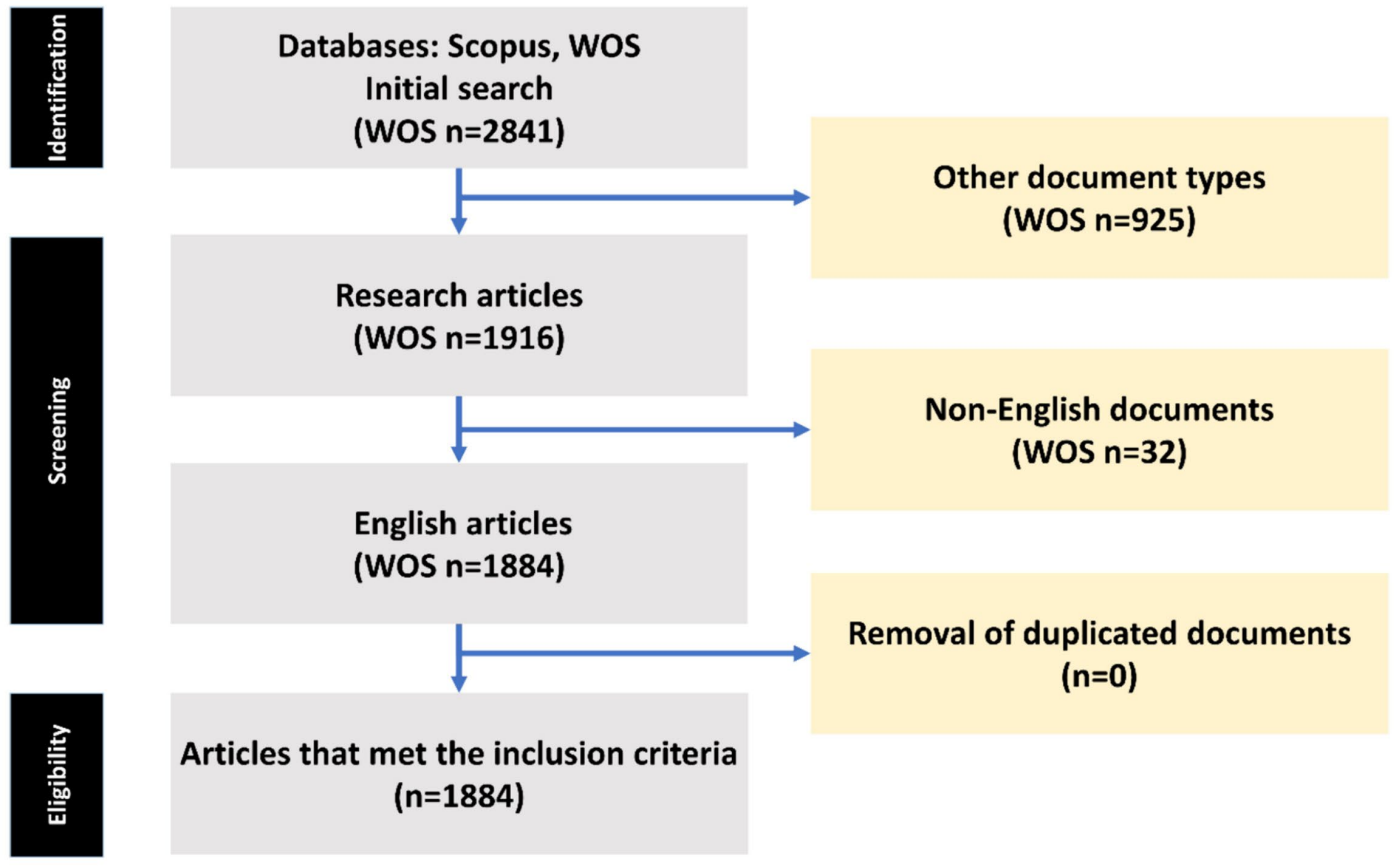
The process of article selection from the Web of Science (WOS) databases is depicted in a flow chart utilizing PRISMA.

To carry out a thorough review of studies focusing on intestinal microbiota in insulin resistance patients, we sourced data from the Web of Science Core Collection (WOS-CC). Our research strategy was devised to be all-encompassing, touching on various aspects of this research area. The process of data gathering took place in May 2024. The search protocol was defined as follows: “Gastrointestinal Microbiome” AND “Insulin Resistance” (Title) or “Gastrointestinal Microbiome” AND “Insulin Resistance” (Abstract) (Table 1). The search covered the years from 2000 to 2024, with no time constraints, in accordance with the indexing in the Web of Science (WOS) database. Our selection criteria were confined to original and review research articles published in English. We ruled out other types of publications and papers not related to the theme of intestinal microbiota in insulin resistance patients. The article selection procedure is demonstrated in Figure 1.

**Table 1:** Web of science database (author keywords OR title OR abstract).

No	Queries
#1	“Gastrointestinal Microbiome” OR “Gastrointestinal Microbiomes” OR “Microbiome, Gastrointestinal” OR “Gut Microbiome” OR “Gut Microbiomes” OR “Microbiome, Gut” OR “Gut Microflora” OR “Microflora, Gut” OR “Gut Microbiota” OR “Gut Microbiotas” OR “Microbiota, Gut” OR “Gastrointestinal Flora” OR “Flora, Gastrointestinal” OR “Gut Flora” OR “Flora, Gut” OR “Gastrointestinal Microbiota” OR “Gastrointestinal Microbiotas” OR “Microbiota, Gastrointestinal” OR “Gastrointestinal Microbial Community” OR “Gastrointestinal Microbial Communities” OR “Microbial Community, Gastrointestinal” OR “Gastrointestinal Microflora” OR “Microflora, Gastrointestinal” OR “Gastric Microbiome” OR “Gastric Microbiomes” OR “Microbiome, Gastric” OR “Intestinal Microbiome” OR “Intestinal Microbiomes” OR “Microbiome, Intestinal” OR “Intestinal Microbiota” OR “Intestinal Microbiotas” OR “Microbiota, Intestinal” OR “Intestinal Microflora” OR “Microflora, Intestinal” OR “Intestinal Flora” OR “Flora, Intestinal” OR “Enteric Bacteria” OR “Bacteria, Enteric”
#2	“Insulin Resistance” OR “Resistance, Insulin” OR “Insulin Sensitivity” OR “Sensitivity, Insulin”
#3	#1 AND #2

18/5/2024.

### 2.2 R studio and Biblioshiny analysis

The bibliometric study was performed using Rstudio v.4.4.0 software, a robust open-source statistical software, along with the bibliometric R-package. This was accessed on the 3rd of May 2024, in tandem with the Biblioshiny tool. Biblioshiny offers an interactive and user-friendly platform for bibliometric studies, facilitating the examination of publication trends, collaboration networks, and keyword linkages (22). Networks of co-authorship, co-occurrence analysis of keywords and citation patterns were formed to uncover collaboration patterns among researchers and influence of articles in this domain.

### 2.3 Delineation of predominant institutions, publications, authors, and allied nations

We formulated visual depictions to showcase the links among the most dynamic institutions and authors, providing a window into their collaborative initiatives. Countries were appraised based on their scholarly output, determined by the share of papers each accounted for. Moreover, we probed the extent of cooperation among the top 10 most industrious nations. To visually encapsulate this cooperation, we designed a map mirroring the count of publications from each country.

### 2.4 Investigation of keyword recurrences

A chronological analysis was performed with enhanced precision to trace the intermittent surfacing of distinct keywords over the years. A TreeMap was assembled to exhibit the spread and salience of the top 10 most recurrent keywords. An exhaustive thematic analysis was undertaken to spotlight the prevailing trends and motifs within the selected articles.

## 3 Results

### 3.1 Comprehensive overview of the manuscripts

The objective of this investigation was to conduct an exhaustive analysis of the worldwide scholarly contributions on relationship between intestinal microbiota and insulin resistance in patients, encompassing articles disseminated from 2000 to 2024. An aggregate of 1884 pertinent studies were meticulously scrutinized, emanating from 510 distinct sources. The examination incorporated the inputs of 12,491 researchers, collectively yielding a commendable mean of 51.36 citations per manuscript over the preceding decades. The salient discoveries pertaining to intestinal microbiota relation with insulin resistance are delineated based on the most frequently cited documents over the previous decades in Table 2. Furthermore, the Annual Growth Rate for this area of research was computed to be 22.08%, signifying a consistent augmentation in publications throughout the duration of the study. The considerable magnitude of research output is further underscored by the incorporation of 63,151 references and 3,022 distinctive author keywords. An overwhelming proportion of authors engaged in cooperative studies (24.95%).

**Table 2:** The 10 most cited documents on the relationship between gut microbiota and insulin resistance (2000–2024).

Rank	Study ID [References]	Title of the document	Journal name	Total citations	DOI/PMID
1	Bäckhed et al. (23)	The gut microbiota as an environmental factor that regulates fat storage	Proceedings of the National Academy of Sciences of the United States	4,298	10.1073/pnas.0407076101
2	Everard et al. (24)	Cross-talk between *Akkermansia muciniphila* and intestinal epithelium controls diet-induced obesity	Proceedings of the National Academy of Sciences of the United States	3,022	10.1073/pnas.1219451110
3	Le Chatelier et al. (25)	Richness of human gut microbiome correlates with metabolic markers	Nature	3,020	10.1038/nature12506
4	Vrieze et al. (26)	Transfer of intestinal microbiota from lean donors increases insulin sensitivity in individuals with metabolic syndrome	Gastroenterology	1966	10.1053/j.gastro.2012.06.031
5	Buzzetti et al. (27)	The multiple-hit pathogenesis of non-alcoholic fatty liver disease (NAFLD)	Metabolism	1791	10.1016/j.metabol.2015.12.012
6	Vijay-Kumar et al. (28)	Metabolic syndrome and altered gut microbiota in mice lacking toll-like receptor 5	Science	1,532	10.1126/science.1179721
7	Pedersen et al. (29)	Human gut microbes impact host serum metabolome and insulin sensitivity	Nature	1,286	10.1038/nature18646
8	Plovier et al. (30)	A purified membrane protein from *Akkermansia muciniphila* or the pasteurized bacterium improves metabolism in obese and diabetic mice	Nature Medicine	1,243	10.1038/nm.4236
9	Dao et al. (31)	*Akkermansia muciniphila* and improved metabolic health during a dietary intervention in obesity: relationship with gut microbiome richness and ecology	Gut	1,183	10.1136/gutjnl-2014-308778
10	Depommier et al. (32)	Supplementation with *Akkermansia muciniphila* in overweight and obese human volunteers: a proof-of-concept exploratory study	Nature Medicine	1,141	10.1038/s41591-019-0495-2

### 3.2 Evolution of publication and citation metrics

**Figure 2:**
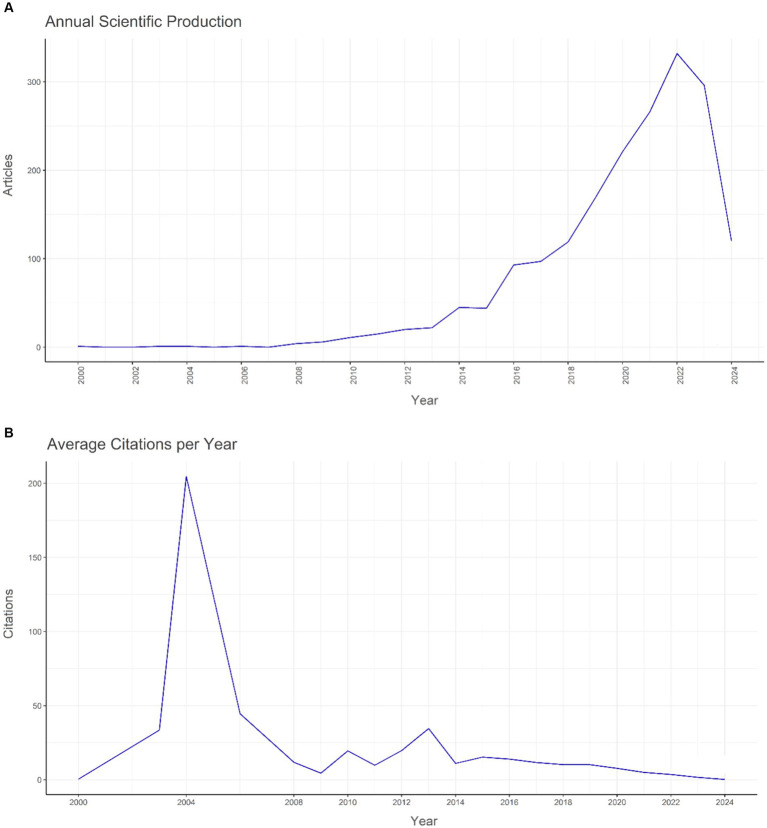
The yearly global pattern of **(A)** publication and **(B)** citation regarding the relationship between intestinal microbiota and insulin resistance from 2000 to 2024.

The dataset reveals oscillations in the average cumulative citations per manuscript over the timeline. The year 2004 witnessed a significant surge in the average cumulative citations per manuscript, peaking at 204.7, whereas 2024 registered the minimum value of 0.2. Moreover, the quantity of publications (N) demonstrated annual variations, with the apex of article production occurring in 2022 (*N* = 332) and the nadir in 2000, 2003, 2004, and 2006 (*N* = 1). The temporal progression of publication is graphically represented in Figure 1 (Figure 2).

**Figure 3:**
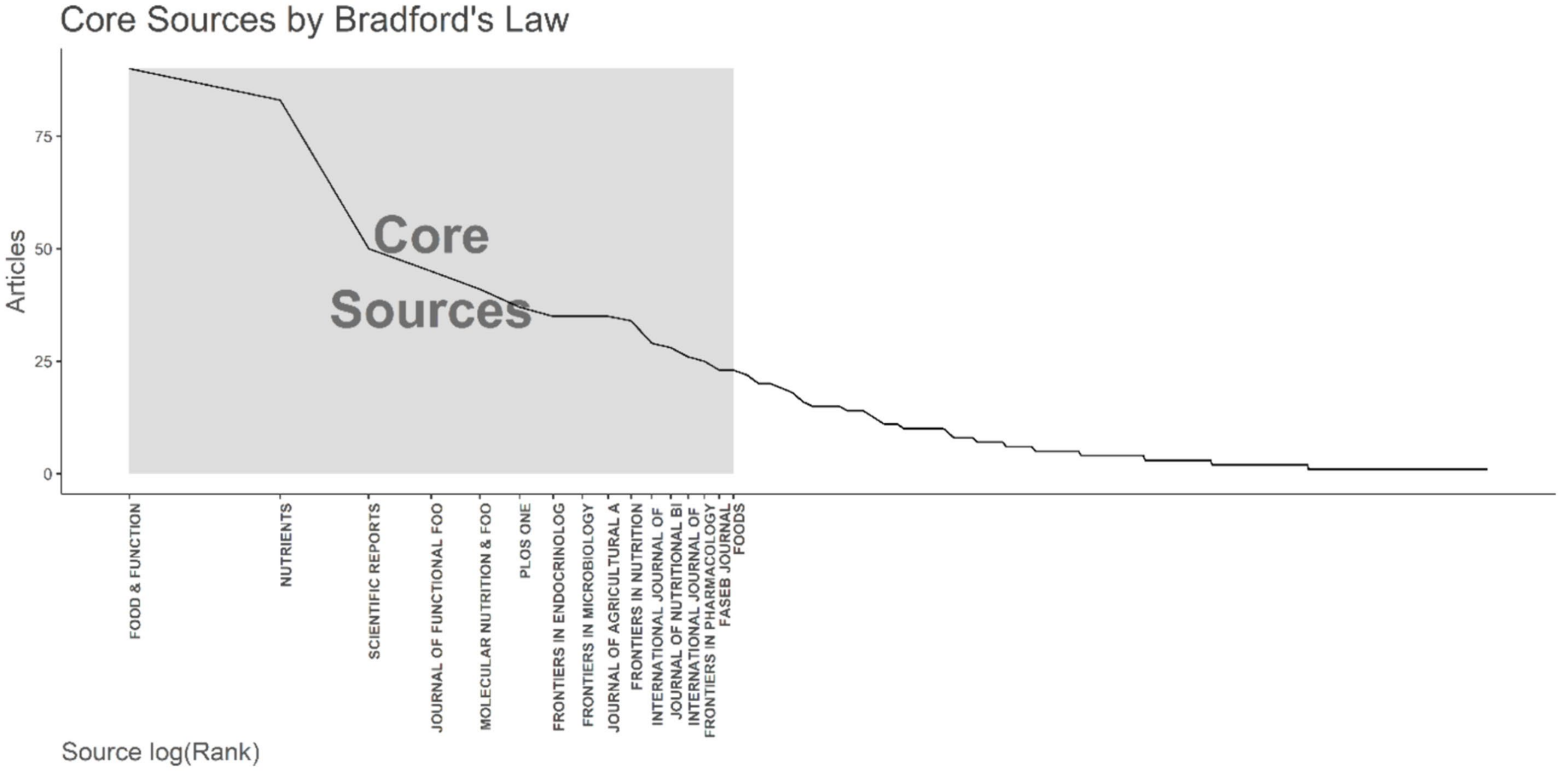
The application of Bradford’s Law is demonstrated, which pinpointed 16 core journals on the topic of the relationship between intestinal microbiota and insulin resistance from 2000 to 2024.

By applying Bradford’s Law, which delineates the dispersion of scholarly articles across various journals, we discerned 16 principal journals that were considered the premier choices for investigators (Figure 3). As per Bradford’s Law, these principal journals collectively constituted a substantial fraction of the aggregate number of articles published on relationship between intestinal microbiota and insulin resistance. Upon scrutinizing the publication data from these principal journals, we noted that the journal “Food & Function” surfaced as the most productive one, contributing a significant 90 articles, which equates to approximately 4.6% of the total articles within the study period. Additionally, we probed into the local citations garnered by these principal journals from other articles within our dataset. Notably, “Nature” distinguished itself with the highest number of local citations, accumulating an impressive total of 3,716 citations (Table 3).

**Table 3:** The 10 most cited journals on the topic of the relationship between gut microbiota and insulin resistance (2000–2024).

Journal	Number of articles
Nature	3,716
PLoS One	2,640
Diabetes	2,479
Proceedings of the National Academy of Sciences of the United States	1950
Nutrients	1839
Gut	1777
Cell Metabolism	1,551
Scientific Reports	1,516
Science	1,353
Journal of Agricultural and Food Chemistry	1,344

### 3.3 Most productive authors, institutions, countries, and their collaboration network

**Figure 4:**
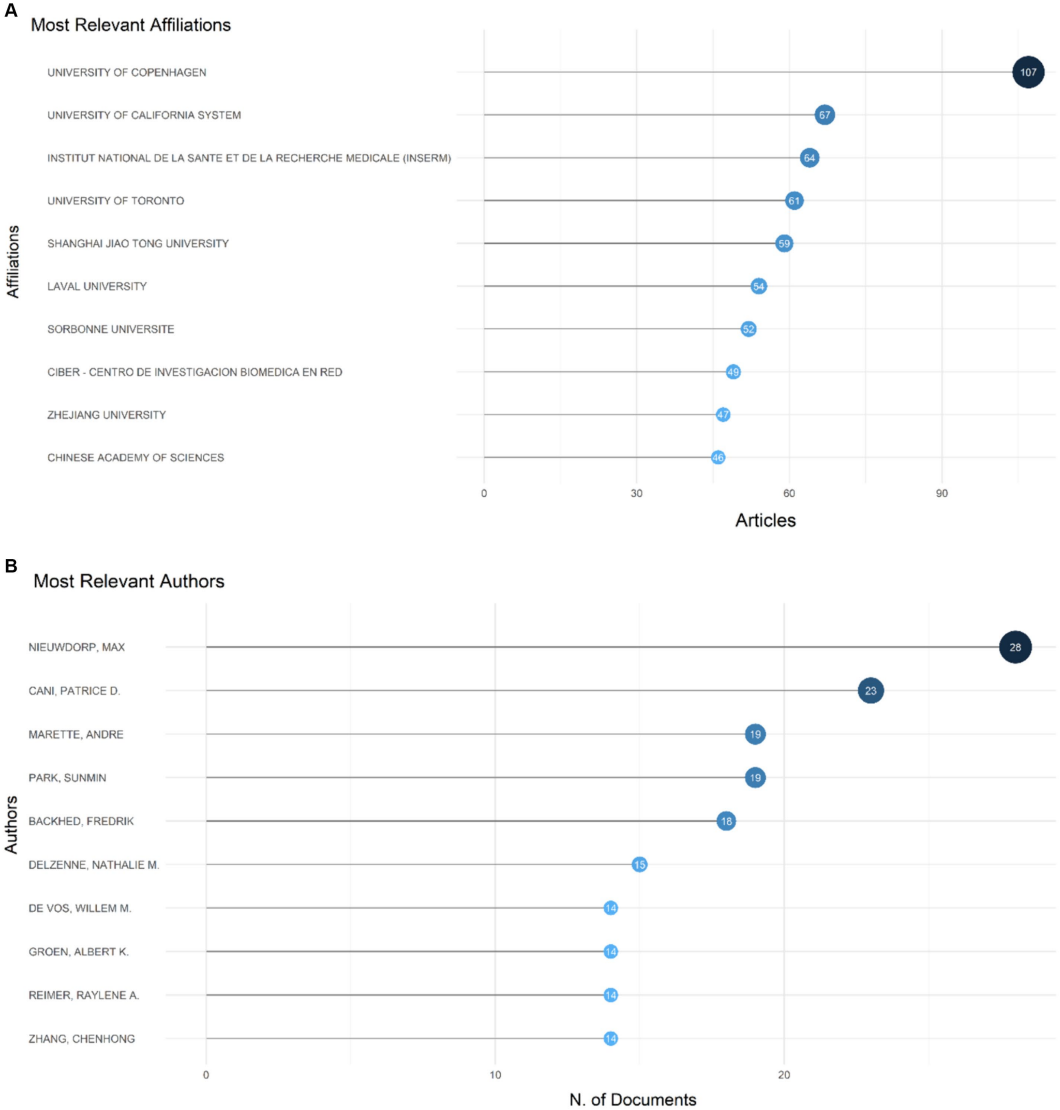
It displays the most prolific authors, institutions, countries, and their collaboration network **(A)**. It also showcases the 10 authors who have made the most significant contributions to the field and their production timeline on the topic of the relationship between intestinal microbiota and insulin resistance from 2000 to 2024 **(B)**.

**Figure 5:**
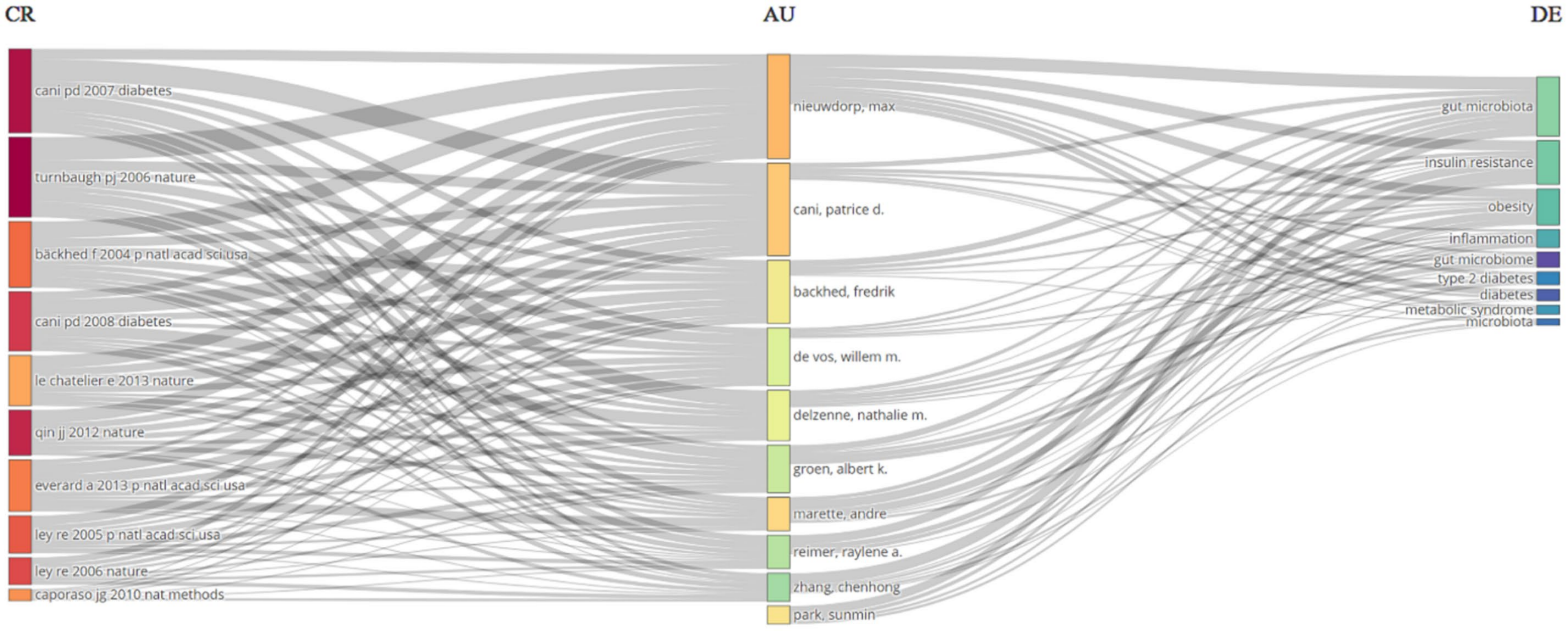
A Three-Fields Plot provides a visual representation of the interactions between cited references (CR), authors (AU), and author keywords (DE) related to the relationship between intestinal microbiota and insulin resistance from 2000 to 2024.

University of Copenhagen, University of California System, Institut National De La Sante Et De La Recherche Medicale (INSERM), University of Toronto, Shanghai Jiao Tong University, Laval University, Sorbonne Universite, Ciber-Centro De Investigacion Biomedica En Red, Zhejiang University, Chinese Academy of Sciences surfaced as the most productive institutions, contributing the highest number of articles with 107, 67, 64, 61, 59, 54, 52, 49, 47, and 46 articles, respectively (Figure 4A). Among the authors, Nieuwdorp M. distinguished himself with the highest number of articles (28, 0.17%), followed by Cani P. D. who produced 23 articles (Figure 4B). The Three-Fields Plot vividly delineates the intricate network of connections among cited references, authors, and author keywords, providing invaluable insights into the complex landscape of relationship between intestinal microbiota and insulin resistance during decades spanning from 2000 to 2024 (Figure 5).

**Figure 6:**
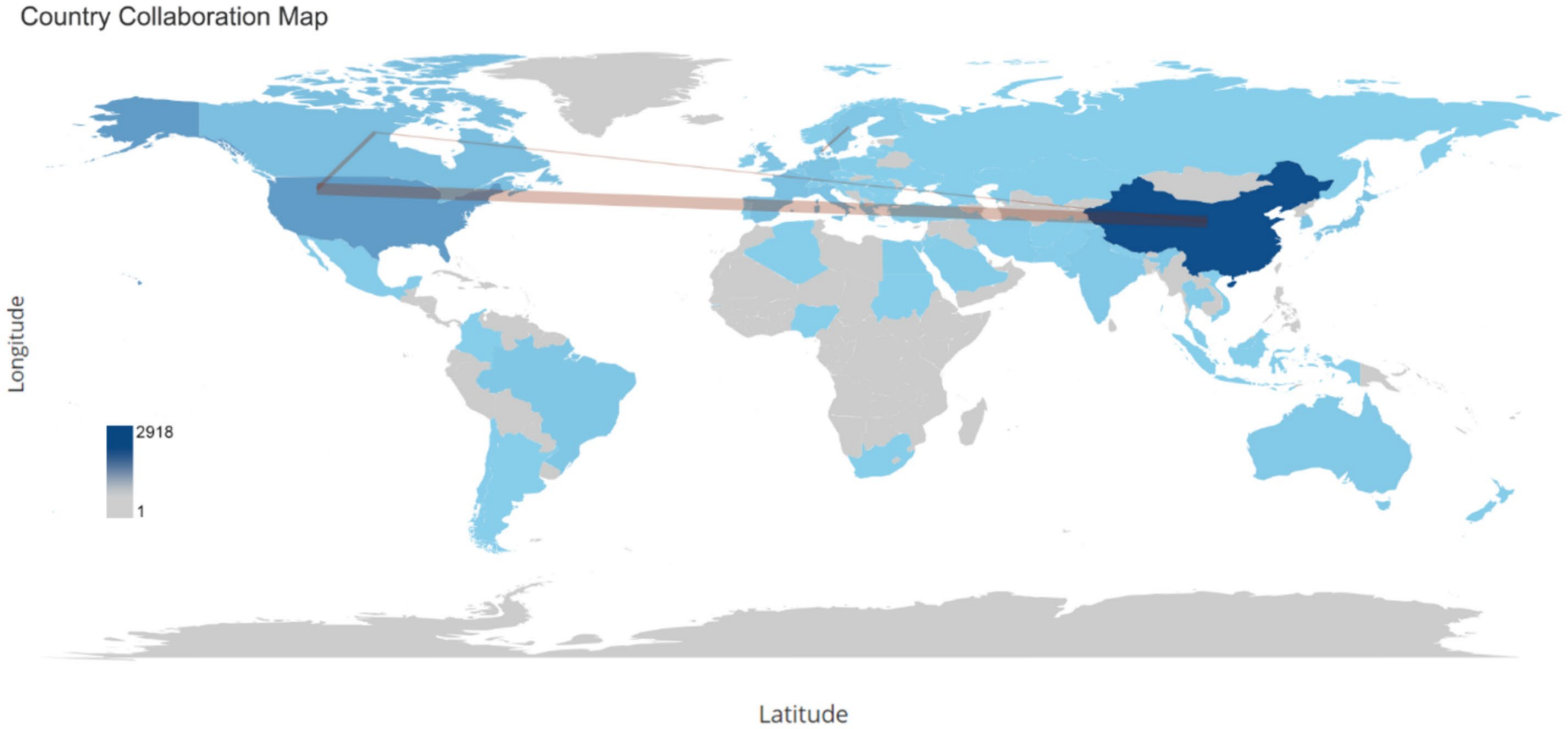
This word map serves as a global collaboration map on the topic of the relationship between intestinal microbiota and insulin resistance from 2000 to 2024. The intensity of color saturation corresponds to the increasing number of articles within each country. The collaboration between countries is symbolized by the thickness of the connecting arrows.

Over the span of decades, China spearheaded the scientific production among countries with 2,918 publications and at the second place the United States had 991publications, followed by Spain with 428 publications, Canada with 348, and Japan with 289 (Table 3). With respect to publication patterns, China exhibited a significant inclination for single-country productions, with 91.9% of its publications. Similarly, the United States demonstrated a high rate of single-country publications at 80.6%. Moreover, China and United States also had highest number of publications collaborated with other countries with 236 and 192 publications. Collaboration strength was predominantly derived from the China and United States, and European countries (Figure 6).

### 3.4 Co-occurrence, focal points, and evolving keywords

**Figure 7:**
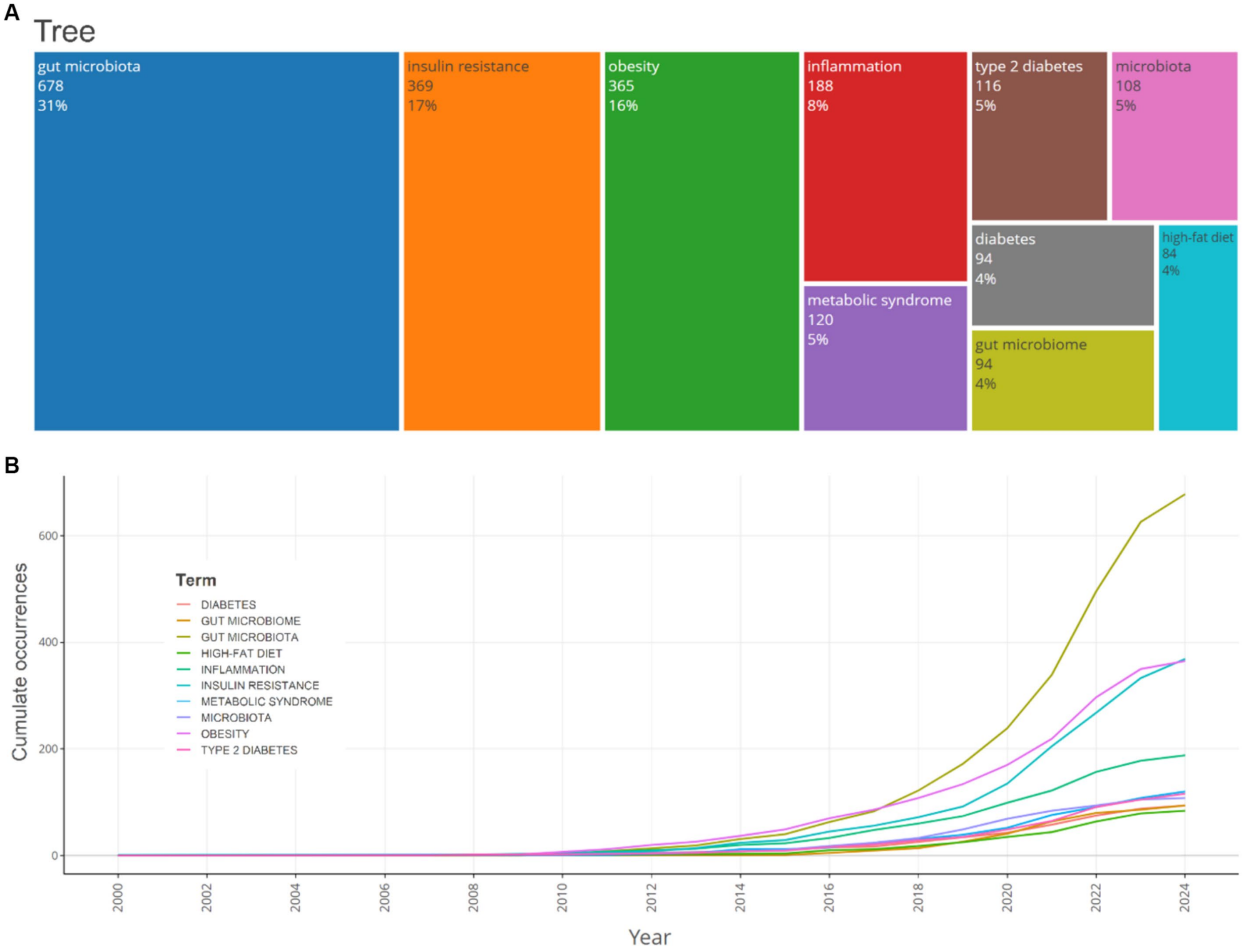
A TreeMap **(A)** and a scatter plot **(B)** that represent the top 10 author’s keywords in the research on the relationship between intestinal microbiota and insulin resistance from 2000 to 2024.

The most frequently encountered author keywords were scrutinized utilizing Biblioshiny. The analysis encompassed commonly observed insulin resistance, gut microbiota, gut microbiome, obesity, and other terminologies associated with insulin resistance and gut microbiota. Keywords pertinent to the subject “gut microbiota,” “insulin resistance” and “obesity” demonstrated an ascending trend, with 678, 369 and 365 instances in 2024, respectively. The frequency of the keyword “high-fat diet” remained relatively constant over the years, with 84 instances in 2024. Moreover, as this study aimed to evaluate the mechanisms of gut microbiota in relation to insulin resistance, the relevant keywords were also assessed. Keywords such as inflammation, chain fatty acids, acid, acids (for bile acids), fatty liver disease, diabetes-mellitus, type-2 diabetes-mellitus, and branched-chain 397, 133, 66, 59, 63, 31, 27, 4 and times in the publications, respectively. Furthermore, keywords related to the treatment of insulin resistance through its relationship with gut microbiota, such as diet and probiotics, prebiotics, and physical-activity appeared 145, 61, 33, and 16 times, respectively (Figure 7).

**Figure 8:**
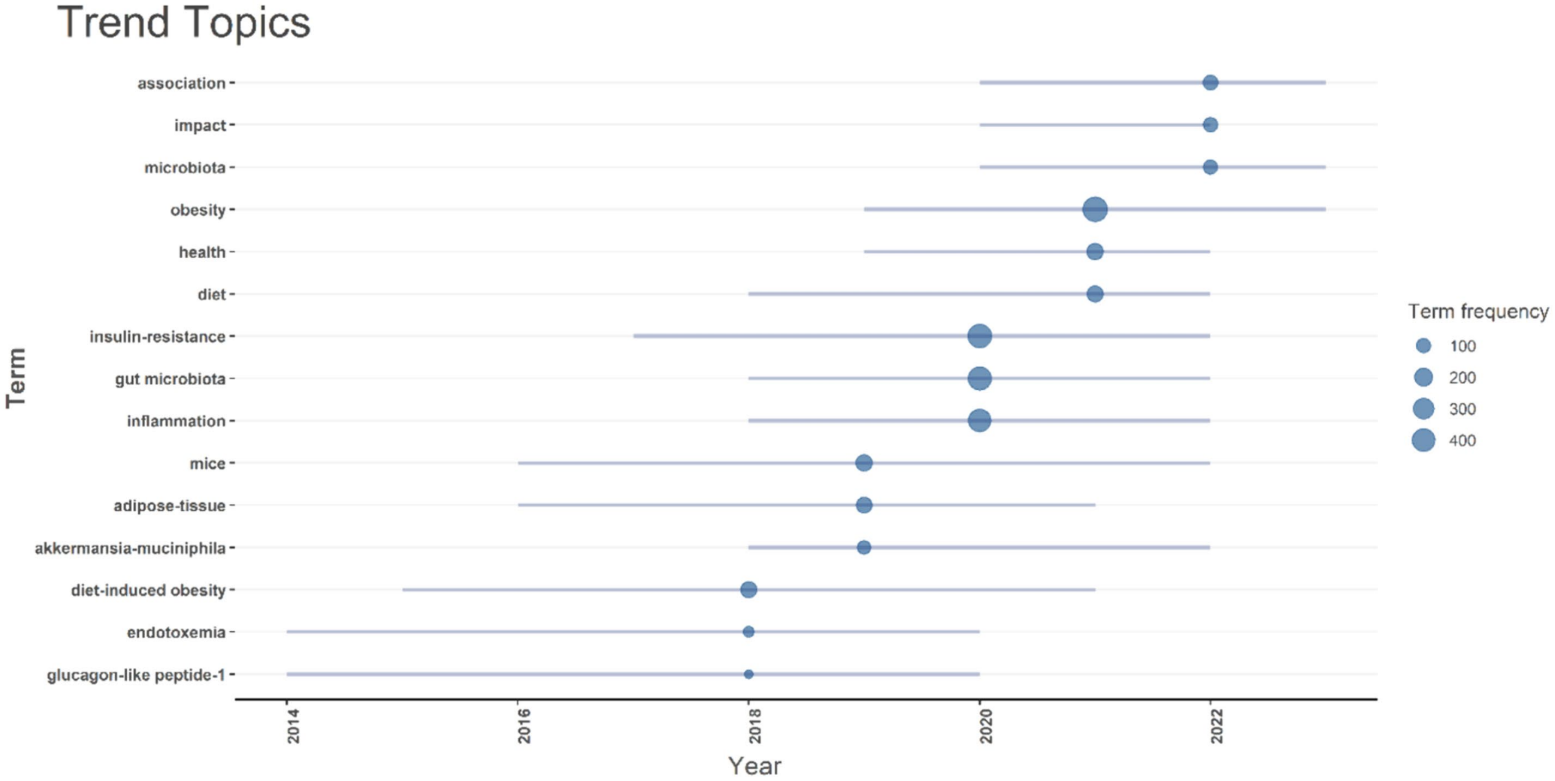
A timeline of trending topics is presented. Each bubble indicates the peak frequency of use for each keyword, while the line indicates the years it was used.

The timeline analysis of crucial keywords reveals that “obesity” and “insulin resistance” attained peak citations in 2021 and 2020, respectively (15.2 and 14%, respectively). Topics such as Gut microbiota (13.2%) and inflammation (12.2%) also possess substantial frequencies, demonstrating the sustained interest in comprehending the relationship between insulin resistance and obesity, and gut microbiota (Figure 8).

## 4 Discussion

### 4.1 Bibliometrics findings

A bibliometric study is fundamentally rooted in the thorough examination of bibliographic data from publications. This encompasses elements like the affiliations of authors, the types of publications, the countries of origin, information on funding, and citation details. In today’s world of learning, there’s been a clear speed-up in areas like bibliometric analysis and scientific mapping. This is mostly because the scientific community is becoming more and more interested in the knowledge gained from different bibliometric studies (33).

The findings of this comprehensive analysis underscore the significant role of gut microbiota in the development of insulin resistance, corroborating previous studies that have highlighted the intricate relationship between intestinal microbiota and metabolic diseases. The substantial body of research reviewed in this study aligns with earlier investigations that have identified gut microbiota as a critical factor influencing insulin resistance and related metabolic disorders.

The consistent annual growth rate of 22.08% in publications related to gut microbiota and insulin resistance underscores the increasing recognition of this field’s importance. The considerable magnitude of research output is further underscored by the incorporation of 63,151 references and 3,022 distinctive author keywords. An overwhelming proportion of authors engaged in cooperative studies (24.95%), highlighting the collaborative nature of this research area. The identification of key journals and prolific authors, such as “Food & Function” and Nieuwdorp M., respectively, highlights the central hubs of research activity and the leading contributors to this domain. This concentration of research efforts in specific journals and by particular authors suggests a robust and collaborative scientific community dedicated to unraveling the complexities of gut microbiota’s role in metabolic health.

The significant contributions from institutions like the University of Copenhagen and the University of California System further emphasize the global nature of this research. Over the span of decades, China spearheaded the scientific production among countries with 2,918 publications, followed by the United States with 991 publications. China exhibited a significant inclination for single-country productions, with 91.9% of its publications. Similarly, the United States demonstrated a high rate of single-country publications at 80.6%. Moreover, China and the USA also had the highest number of publications collaborated with other countries, with 236 and 192 publications, respectively. Collaboration strength was predominantly derived from China, the United States, and European countries.

The analysis of author keywords revealed that terms such as “gut microbiota,” “insulin resistance,” and “obesity” demonstrated an ascending trend, with 678, 369, and 365 instances in 2024, respectively. The frequency of the keyword “high-fat diet” remained relatively constant over the years, with 84 instances in 2024. The timeline analysis of crucial keywords reveals that “obesity” and “insulin resistance” attained peak citations in 2021 and 2020, respectively (15.2 and 14%, respectively). Topics such as gut microbiota (13.2%) and inflammation (12.2%) also possess substantial frequencies, demonstrating the sustained interest in comprehending the relationship between insulin resistance, obesity, and gut microbiota.

### 4.2 Limitations of bibliometrics study

Although bibliometric methods offer a quantitative snapshot of research trends, they contribute minimally to the literature because they emphasize publication metrics over the deep conceptual understanding of the topic (34). These approaches tend to highlight citation counts and publication volumes, which can obscure significant scholarly contributions such as theoretical advancements and methodological innovations (35). As a result, bibliometric studies often overlook critical aspects of research content, such as the robustness of methods, the quality of evidence, and theoretical contributions, all essential for a thorough evaluation of scientific progress (36). This focus on quantitative metrics can also introduce biases, as highly cited research is not always the most innovative or impactful (37). Moreover, bibliometric methods frequently fail to capture the value of interdisciplinary research accurately, thus neglecting significant contributions that do not conform to traditional citation databases (38). Therefore, while these methods are helpful for identifying general patterns, they should be combined with qualitative assessments to gain a comprehensive understanding of research impact and quality (39).

In order to overcome these limitations, the current study performed a comprehensive literature review that significantly enriches the manuscript’s contribution.

### 4.3 Comparison with previous literature

The role of gut microbiota in metabolic diseases, particularly insulin resistance, has been extensively documented in the literature. Caricilli and Saad (6) emphasized that gut microbiota contributes to metabolic diseases by influencing low-grade inflammation, a key mechanism in insulin resistance (6, 40). This study’s findings are consistent with their assertion, as the reviewed articles frequently cited inflammation as a pivotal factor in the gut microbiota-insulin resistance axis.

Moreover, the transplantation of healthy intestinal microbiota into germ-free mice resulted in increased body fat and insulin resistance, establishing a direct link between gut microbiota and metabolic health (41). This foundational study is supported by the current analysis, which shows a consistent increase in publications exploring this relationship, reflecting the growing scientific interest and validation of these early findings.

The review by Bastos et al. (13) further elucidated the role of gut-microbiota-derived metabolites, such as SCFAs, in modulating insulin signaling pathways (12, 13). The present study’s identification of frequently encountered keywords like “SCFAs” and “gut microbiota” aligns with these findings, indicating a sustained research focus on these metabolites and their impact on insulin resistance.

### 4.4 Mechanisms of gut microbiota interference with insulin resistance

The results of the current study’s keyword analysis revealed important terms related to the mechanisms of gut microbiota interference with insulin resistance. The keywords inflammation, chain fatty acids, acid, acids (for bile acids), and fatty liver disease showed a high frequency among previous publications. Consequently, the next step of the current study focused on the detailed mechanisms of gut microbiota interference with insulin resistance. The mechanisms by which gut microbiota influence insulin resistance are multifaceted and involve several pathways. One critical mechanism is the alteration of gut permeability and the subsequent increase in systemic inflammation. Studies have shown that an increased proportion of Firmicutes and Actinobacteria, along with a decreased proportion of Bacteroidetes, are associated with higher serum lipopolysaccharide levels, which trigger inflammatory pathways and impair insulin signaling (6). Additionally, gut microbiota-derived metabolites, such as SCFAs, play a significant role in modulating host metabolism. This result aligns with the bibliometric analysis of the current study, which also showed a high frequency of these terms, indicating the significance of this mechanism. SCFAs like butyrate and acetate are known to enhance insulin sensitivity by activating G-protein-coupled receptors and inhibiting histone deacetylases (HDACs), which in turn regulate gene expression related to glucose and lipid metabolism (5). Furthermore, the gut microbiota influences bile acid metabolism, which affects glucose homeostasis and energy expenditure through the activation of farnesoid X receptor and Takeda G-protein-coupled receptor 5 (42). Moreover, the gut microbiota can influence the integrity of the intestinal barrier. A compromised barrier allows the translocation of lipopolysaccharides (LPS) from gram-negative bacteria into the bloodstream, triggering an inflammatory response that impairs insulin signaling. Chronic low-grade inflammation, mediated by pro-inflammatory cytokines such as TNF-α and IL-6, has been implicated in the pathogenesis of insulin resistance (43–45).

### 4.5 Metabolite production

As previously mentioned, due to the high frequency of SCFA and its important role in the gut microbiota and insulin resistance, additional metabolites of the gut microbiota related to insulin resistance were also evaluated. However, in the author’s keywords from the bibliometric analysis of the current study, a few keywords related to these metabolites, such as branched-chain amino acids (BCAAs), were found. Despite this, the current study discusses these metabolites due to their importance. In detail, gut microbiota produces various metabolites, including SCFAs, bile acids, and branched- BCAAs, which play crucial roles in metabolic regulation (46, 47). SCFAs like butyrate and propionate have been shown to enhance insulin sensitivity by activating G-protein-coupled receptors (GPR41 and GPR43) and inhibiting histone deacetylases (HDACs) (48). Conversely, elevated levels of BCAAs have been associated with insulin resistance, potentially due to their influence on mTOR signaling and the promotion of inflammatory pathways (49).

### 4.6 Detailed mechanisms

#### 4.6.1 Short-chain fatty acids

SCFAs, primarily acetate, propionate, and butyrate, are produced by the fermentation of dietary fibers by gut bacteria. These metabolites play significant roles in host energy homeostasis and insulin sensitivity (50–52). Butyrate serves as a primary energy source for colonic epithelial cells and enhances gut barrier function, reducing endotoxemia and systemic inflammation. SCFAs also activate G-protein-coupled receptors (GPR41 and GPR43), which influence glucose and lipid metabolism, and inhibit HDACs, thereby regulating gene expression involved in insulin signaling (52, 53).

#### 4.6.2 Bile acids

Another substance produced by the gastrointestinal system is bile acids. The results of the current study showed that author keywords related to bile acids, such as acid and acids, had a relatively high frequency. This provides further evidence that metabolites of the gastrointestinal system play an important role in insulin resistance and are prominent in the literature. Consistent with the findings of the current study, previous studies have also highlighted the importance of bile acids. Gut microbiota modulate the composition of bile acids, which are critical regulators of glucose and lipid metabolism (54). Primary bile acids synthesized in the liver are converted into secondary bile acids by gut bacteria (55). These secondary bile acids can activate the farnesoid X receptor (FXR) and the Takeda G-protein-coupled receptor 5 (TGR5), both of which play roles in maintaining metabolic homeostasis (56). FXR activation improves insulin sensitivity and reduces hepatic gluconeogenesis, while TGR5 activation stimulates GLP-1 secretion, enhancing insulin secretion and sensitivity (57).

#### 4.6.3 Branched-chain amino acids

As previously mentioned, BCAAs had very low frequency among the author keywords in this bibliometric study. However, in contrast to this finding, some previous literature has demonstrated the important role of BCAAs in relation to insulin resistance. Elevated levels of BCAAs have been associated with insulin resistance and metabolic disorders. Gut microbiota contribute to the metabolism of BCAAs, and dysbiosis can lead to altered BCAA levels. BCAAs may impair insulin signaling through the activation of mTOR pathways and promote inflammation by stimulating pro-inflammatory cytokine production. Targeting BCAA metabolism through dietary interventions or modulation of gut microbiota offers a potential therapeutic approach for improving insulin sensitivity (47, 58). Despite the importance of BCAAs in insulin resistance, the low frequency of this author keyword suggests that this component needs to be more evaluated in future studies.

### 4.7 Relationship to obesity and other related diseases

The interplay between gut microbiota, insulin resistance, and obesity is well-documented, with evidence suggesting that alterations in gut microbiota contribute to the development of obesity and related metabolic disorders such as type 2 diabetes (T2D) and non-alcoholic fatty liver disease (NAFLD) (59, 60). The results of the current study align with the literature, which shows the high frequency of author keywords such as obesity, diabetes mellitus, type 2 diabetes mellitus, and fatty liver acids. This underscores the importance of these keywords and their mechanisms of action in insulin resistance. In subsequent paragraphs, the current study will explore these keywords and their relationship with insulin resistance in more detail.

#### 4.7.1 Obesity

Obesity is associated with significant changes in gut microbiota composition, including a reduced diversity and an altered ratio of *Firmicutes* to *Bacteroidetes* (61). These changes can lead to increased energy harvest from the diet, promoting weight gain and adiposity (62, 63). Additionally, gut microbiota can influence the host’s lipid metabolism and adipose tissue function, further contributing to obesity (62–64).

#### 4.7.2 Type 2 diabetes

Insulin resistance is a hallmark of T2D, and gut microbiota have been implicated in its pathogenesis. Dysbiosis can exacerbate insulin resistance through mechanisms involving inflammation, altered gut permeability, and metabolic endotoxemia. Moreover, gut microbiota-derived metabolites such as SCFAs and secondary bile acids can modulate glucose metabolism and insulin sensitivity (59, 60).

#### 4.7.3 Non-alcoholic fatty liver disease

NAFLD is characterized by excessive fat accumulation in the liver and is closely linked to obesity and insulin resistance (65, 66). Gut microbiota contribute to NAFLD through several mechanisms, including the modulation of hepatic lipid metabolism, inflammation, and the gut-liver axis (67, 68). Dysbiosis can lead to increased intestinal permeability, facilitating the translocation of LPS and other bacterial products that promote hepatic inflammation and fibrosis (66, 69).

### 4.8 Treatment methods

#### 4.8.1 Dietary interventions

The results of the current bibliometric study showed that the author keyword “diet” had a high frequency with 145 instances. This highlights the importance of dietary interventions in previous publications. Consistent with this finding, previous studies have also demonstrated the significance of dietary interventions. Previous studies showed, modifying diet to alter gut microbiota composition and function has shown promise in improving insulin sensitivity and metabolic health (42, 70). Diets rich in fibers, such as the Mediterranean diet, promote the production of beneficial SCFAs and enhance gut barrier function (70, 71). Conversely, high-fat and high-sugar diets can promote dysbiosis and metabolic endotoxemia, exacerbating insulin resistance (71, 72).

Efficacy: High efficacy in improving metabolic health, particularly when combined with other lifestyle changes. Promotes long-term benefits by addressing the root cause of dysbiosis and metabolic dysregulation (42, 71).

#### 4.8.2 Probiotics and prebiotics

Probiotics and prebiotics both had a considerable frequency among publications. However, probiotics were mentioned nearly twice as often as prebiotics. The significance of these supplements has also been demonstrated in previous studies. Probiotics, live beneficial bacteria, and prebiotics, non-digestible food components that stimulate the growth of beneficial bacteria, have been used to modulate gut microbiota (73). Clinical trials have demonstrated that probiotics can improve insulin sensitivity and reduce inflammation in individuals with metabolic disorders (74, 75). Prebiotics, such as inulin and fructooligosaccharides, promote the growth of SCFA-producing bacteria, enhancing metabolic health (76).

Efficacy: Effective in improving insulin sensitivity and reducing inflammation, with varying results depending on the strains used and individual patient characteristics (77).

#### 4.8.3 Fecal microbiota transplantation

This term, fecal microbiota transplantation was not observed in the author keywords result of this study; however, this new method showed positive effects on treatment of insulin resistance in previous studies. FMT involves the transfer of stool from a healthy donor to the gut of a recipient to restore a healthy microbiota composition (78). This treatment has shown potential in treating metabolic disorders, including insulin resistance and obesity (79). Studies have reported improvements in insulin sensitivity and metabolic profiles following FMT (80). However, the long-term efficacy and safety of FMT require further investigation (81).

Efficacy: Promising results in clinical trials, but variability in outcomes and potential risks highlight the need for more research to establish standardized protocols (82).

#### 4.8.4 Pharmacological interventions

The exact term of pharmacological interventions in the bibliometrics analysis of the current study was not found. However, the effects of some drugs are well-known in treatment of insulin resistance. Several pharmacological approaches target gut microbiota to improve metabolic health (83, 84). Antibiotics, though not a long-term solution, have been used to temporarily reduce gut bacterial load and inflammation, leading to improved insulin sensitivity (84, 85). Additionally, bile acid sequestrants, which alter bile acid metabolism, have shown potential in modulating gut microbiota and improving glucose metabolism (54).

Efficacy: Provides rapid improvements in insulin sensitivity but may have limited long-term benefits and potential side effects. Antibiotics, in particular, can disrupt the gut microbiota balance (84, 85).

#### 4.8.5 Physical activity

Another term related to the treatment of insulin resistance is “physical activity,” which appeared 16 times among the keywords in the current study. Although this frequency is not very high, publications related to physical activity have demonstrated its significant efficacy. Exercise is a well-established method to improve insulin sensitivity and metabolic health (86, 87). Physical activity can also modulate gut microbiota composition, increasing the abundance of beneficial bacteria and enhancing SCFA production (88). The MyoGlu clinical trial demonstrated that structured exercise programs could reduce BCAA levels and improve insulin sensitivity, highlighting the multifaceted benefits of physical activity in metabolic regulation (89).

Efficacy: Highly effective in improving insulin sensitivity and overall metabolic health. Offers additional benefits such as weight management and cardiovascular health improvements (90).

### 4.9 Future prospects

Looking forward, the field of gut microbiota and insulin resistance research is poised for further advancements. The increasing use of culture-independent sequencing techniques, as highlighted by Caricilli and Saad (6), will likely continue to uncover new microbial genes and disease-associated patterns, enhancing our understanding of the gut microbiome’s role in metabolic diseases. Additionally, the therapeutic potential of targeting gut-microbiota-derived metabolites, as discussed by Bastos et al. (13), presents a promising avenue for developing novel treatments for insulin resistance and related conditions (12, 13).

Future research should focus on standardizing methodologies to identify and select probiotic strains with proven antidiabetic properties, as suggested by the review in “Frontiers in Endocrinology” (2019) (17, 18). This will be crucial for translating preclinical findings into effective clinical interventions. Moreover, large-scale clinical trials are needed to validate the therapeutic potential of gut microbiota manipulation in humans, ensuring that these strategies can be effectively implemented to combat metabolic diseases on a broader scale.

## 5 Conclusion

This comprehensive bibliometric analysis underscores the pivotal role of gut microbiota in the development of insulin resistance, aligning with previous findings that highlight its influence on metabolic health. The substantial body of literature reviewed reveals a consistent annual growth rate of 22.08%, reflecting the increasing importance of this research area. Key contributors and journals, such as Nieuwdorp M. and “Food and Function,” signify central hubs for ongoing research. The findings suggest that while significant progress has been made, further investigation is necessary to standardize methodologies and explore the long-term therapeutic potential of gut microbiota manipulation in treating insulin resistance and related metabolic disorders. Continued exploration in this field is essential for developing effective interventions and improving metabolic health outcomes globally.

## Data Availability

The original contributions presented in the study are included in the article, further inquiries can be directed to the corresponding author/s.
